# Bridging the Family Care Gap

**DOI:** 10.1093/geroni/igab046.1032

**Published:** 2021-12-17

**Authors:** Joseph Gaugler, Richard Schulz

## Abstract

This symposium aims to create a scientific and policy roadmap to offset the impending shortage of family caregivers available to assist older adults in the U.S. (i.e., the “family care gap”). Drawing on public health, cultural frameworks, family care science, and policy analysis, this symposium will orient future research, intervention development, dissemination and implementation, and policy innovation to more effectively address the family care gap. The selected presentations will include the need to apply and understand cultural adaptation and humility to support a rapidly diversifying older population (Drs. Nkimbeng and Parker). In addition, systematic review methodology will be applied to obtain insights as to what intervention models/strategies actually reduce caregiving time (Drs. Baker, Jutkowitz, and Gaugler). The next presentation will leverage the existing evidence base of translational efforts that aim to disseminate and implement dementia caregiver interventions into practice (Drs. Hodgson and Gitlin). The final presentation of our symposium will focus in-depth on a potential solution to the family care gap: more systematic approaches to identifying and assessing family caregivers in healthcare systems (Drs. Riffin and Wolff). Our discussant, Dr. Richard Schulz, will bring his extensive and renowned experience in caregiving to summarize the public health and policy implications of the family care gap.

